# Prognostic utility of TME-associated genes in pancreatic cancer

**DOI:** 10.3389/fgene.2023.1218774

**Published:** 2023-09-01

**Authors:** Yuanhua Nie, Longwen Xu, Zilong Bai, Yaoyao Liu, Shilong Wang, Qingnuo Zeng, Xuan Gao, Xuefeng Xia, Dongmin Chang

**Affiliations:** ^1^ Department of Surgical Oncology, The First Affiliated Hospital of Xi’an Jiaotong University, Xi’an, Shaanxi, China; ^2^ Geneplus-Beijing, Co., Ltd., Beijing, China; ^3^ State Key Laboratory of Microbial Resources, Institute of Microbiology, Chinese Academy of Sciences, Beijing, China; ^4^ GenePlus- Shenzhen Clinical Laboratory, Shenzhen, China

**Keywords:** pancreatic cancer, tumor microenvironment, prognostic risk model, immunotherapy, therapeutic target

## Abstract

**Background:** Pancreatic cancer (PC) is a deadly disease. The tumor microenvironment (TME) participates in PC oncogenesis. This study focuses on the assessment of the prognostic and treatment utility of TME-associated genes in PC.

**Methods:** After obtaining the differentially expressed TME-related genes, univariate and multivariate Cox analyses and least absolute shrinkage and selection operator (LASSO) were performed to identify genes related to prognosis, and a risk model was established to evaluate risk scores, based on The Cancer Genome Atlas (TCGA) data set, and it was validated by external data sets from the Gene Expression Omnibus (GEO) and Clinical Proteomic Tumor Analysis Consortium (CPTAC). Multiomics analyses were adopted to explore the potential mechanisms, discover novel treatment targets, and assess the sensitivities of immunotherapy and chemotherapy.

**Results:** Five TME-associated genes, namely, *FERMT1*, *CARD9*, *IL20RB*, *MET*, and *MMP3*, were identified and a risk score formula constructed. Next, their mRNA expressions were verified in cancer and normal pancreatic cells. Multiple algorithms confirmed that the risk model displayed a reliable ability of prognosis prediction and was an independent prognostic factor, indicating that high-risk patients had poor outcomes. Immunocyte infiltration, gene set enrichment analysis (GSEA), and single-cell analysis all showed a strong relationship between immune mechanism and low-risk samples. The risk score could predict the sensitivity of immunotherapy and some chemotherapy regimens, which included oxaliplatin and irinotecan. Various latent treatment targets (*LAG3*, *TIGIT*, and *ARID1A*) were addressed by mutation landscape based on the risk model.

**Conclusion:** The risk model based on TME-related genes can reflect the prognosis of PC patients and functions as a novel set of biomarkers for PC therapy.

## Introduction

Pancreatic cancer (PC) is one of the most common and lethal cancers worldwide (Kaźmierczak-Siedlecka et al., 2020). In developed countries, PC is the fourth leading cause of cancer-related deaths, and it is ranked the seventh around the world (Ducreux et al., 2015). Sadly, the incidence rate of PC is gradually accelerating, and it will rank as the second leading cause of cancer-related mortalities in 2030 (Hasan et al., 2019). The treatments for early stage PC are surgery and chemotherapy. The treatments for advanced PC are chemotherapy and radiotherapy (Ducreux et al., 2015). The most used and acknowledged chemotherapy regimens for all stages of PC constitute cytotoxic drugs, such as, FOLFIRINOX and gemcitabine plus nab-paclitaxel (Conroy et al., 2011; Von Hoff et al., 2013). Disappointedly, patients are often resistant to these treatments and tend to have a poor prognosis (Jiang et al., 2023). For the minority of early stage patients with local disease, the 5-year survival rate can reach 36%, and the rate decreases to 12% in patients with lymph-node metastasis. Most patients who suffer from distant spread have the lowest 5-year survival, with 3% (Poruk et al., 2013; Bray et al., 2018). Therefore, it is necessary to find a novel and potent method to perform risk assessment to recognize high-risk patients in the early stage and provide them with proper treatment to avoid cancer progression.

The tumor microenvironment (TME) is an immunosuppressive niche that is formed in the process of tumor cells hijacking the transcriptional mechanisms of the stroma cells (Kleeff et al., 2016). The main components of the TME are cancer-associated fibroblasts (CAFs), extracellular matrix (ECM), endothelial cells, stroma-associated pancreatic stellate cells (PSCs), adipose cells, neural cells, and some immune cells, such as myeloid-derived suppressor cells (MDSCs), tumor-associated macrophages (TAMs), and regulatory T cells (Tregs) (Feig et al., 2012; Wolfgang et al., 2013). PC is notorious for its dense TME, which is enriched with the stroma, MDSCs, TAMs, CAFs, and many other cells (Farrow et al., 2008). As immunosuppressive TME characteristics, MDSCs block immune responses and release interleukin-10 (IL-10) and transforming growth factor-β (TGF-β) to induce an anti-inflammatory environment in PC (Huang et al., 2006; Sinha et al., 2007; Ostrand-Rosenberg et al., 2012; Pinton et al., 2016). CAFs fulfill the bi-function in PC, which is mostly anti-immune and partial immunosuppression (Belle and DeNardo, 2019; Elyada et al., 2019; Das et al., 2020). By promoting the epithelial–mesenchymal transformation (EMT), interacting with cancer stem cells (CSCs), inducing the apoptosis of T cells, and breaking local immune surveillance, the TAMs boost PC, lead to resistance of treatment, and result in poor prognosis (Zhang et al., 2022). Treg cells eliminate effector T cells or acquire antigen-presenting cells which compete with effector T cells against immunology (Jang et al., 2017). However, there are still some immune cells that play anti-tumor roles and offer promising prospects for survival in PC. Tertiary lymphoid structures, organized by tumor-infiltrating lymphocytes (TILs), which are often observed in cancer tissue, are considered to participate in the immune response to suppress cancer and positively impact prognosis (Balch et al., 1990; Zhang et al., 2003; Hiraoka et al., 2015). Infiltration of CD8^+^ lymphocytes was an independent factor for longer disease-free survival (DFS) and overall survival (OS) in PC (Lohneis et al., 2017). The roles of the TME are complex: some can be used to foresee the prognosis, while some are indexes for the sensitivity of immunotherapy of PC (Nomi et al., 2007; Samstein et al., 2019). However, there are no immune markers that can solve well all the problems at one times. Therefore, we established a TME-related risk model to predict survival and to test drug sensitivity in PC.

In this study, the expression patterns of TME-related genes in PC were comprehensively revealed, and we established a new but robust risk model to predict the prognosis, identify therapy targets, and foresee the treatment sensitivity of PC patients.

## Materials and methods

### Gene expression and clinical data resources and processing

**FIGURE 1:**
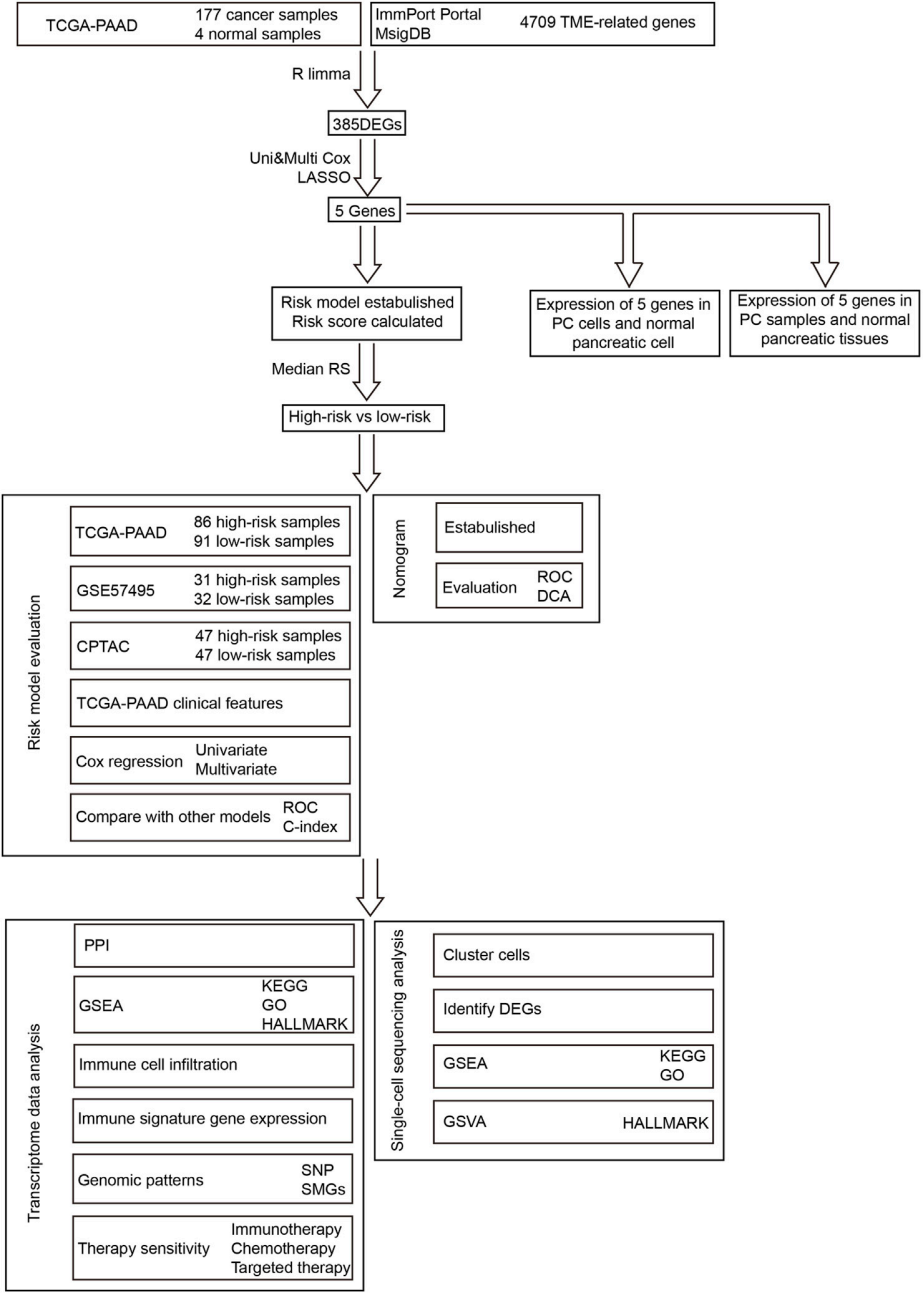
Workflow of this research.

The workflow of our research is presented in Figure 1. To build and evaluate the risk model, PC data sets containing complete information of genome, prognosis, and clinical characteristics were included in this study. The FPKM-processed RNA sequencing data and clinical information of pancreatic adenocarcinoma (PAAD) patients were downloaded from The Cancer Genome Atlas (TCGA, https://tcga-data.nci.nih.gov/tcga/). The RMA-normalized data and clinical data of the PC cohort GSE57495 were obtained from the Gene Expression Omnibus (GEO, https://www.ncbi.nlm.nih.gov/geo/). The RSEM-standardized data from the Clinical Proteomic Tumor Analysis Consortium (CPTAC) were retrieved from the cBioPortal database (https://www.cbioportal.org/). After converting ensemble IDs, deleting the data that lacked survival features, and log2 transforming RNA sequencing, the data were corrected using the combat method (Johnson et al., 2007). The clinical information of TCGA-PAAD, GEO57495, and CPTAC is provided in Supplementary Table S1. The data of single-cell RNA sequencing were retrieved from the cohort GSE141017 via the GEO database. The TCGA-PAAD, GSE57495, CPTAC, and GSE141017 cohorts contained 181 (177 tumor and 4 normal) samples, 63 tumor samples, 135 tumor samples, and 1 tumor sample, respectively.

To evaluate the mRNA expression of five genes in PC and normal pancreatic tissues, data sets containing the mRNA expression of the required genes in normal and cancer tissues were included. We downloaded the RMA-normalized gene expression data of cohorts GSE15471, GSE28735, and GSE62452 from the GEO database. GSE15471, GSE28735, and GSE62452 cohorts contain 78 (39 tumor and 39 normal) samples, 90 (45 tumor and 45 normal) samples, and 130 (65 tumor and 65 normal) samples, respectively. The landscape of data sets which were used in this study is summarized in Supplementary Table S1.

### Identification of differential genes

In total, we collected 4,709 TME-related genes from the ImmPort Portal (https://www.immport.org/) and Molecular Signatures Database (MsigDB, http://www.gsea-msigdb.org/gsea/msigdb/index.jsp) (Supplementary Table S2). The criteria were set as |logFC| >1 and *p* < 0.05 for the expression of the differential genes which were selected by the R package “limma” in normal and cancer samples in TCGA-PAAD.

### Establishment and validation of prognostic risk model

First, the TCGA-PAAD cohort was randomly assigned to train and test the data sets. A prognostic risk model was established based on the training set of the TCGA-PAAD cohort. Then, the univariate Cox regression analysis was used to identify the candidate TME-related genes that were closely correlated with the OS of PC patients, and 86 genes were selected for the next step (*p* < 0.05). To remove the overfit genes, the least absolute shrinkage and selection operator (LASSO) was fulfilled by the R package “glmnet” with a 20-fold cross-validation (Friedman et al., 2010). After LASSO, nine genes turned out to be more reliable nominees for building a prognostic risk model. Next, the Akaike information criterion (AIC) was calculated to assess potential genes. At last, the multivariate Cox regression with a bi-directional method was applied for choosing the best candidates for model construction which presents the minimum AIC. A prognostic risk score model based on the TME-related genes for PC patients was constructed, and the risk score was the sum of the mRNA expression of the gene multiplied by its multivariate Cox regression coefficient (Friedman et al., 2010; Wang et al., 2019). The risk score (RS) of each sample was calculated according to this formula. All samples from the TCGA-PAAD cohort were divided into high- and low-risk groups in accordance with the median value of RS of the TCGA-PAAD training set. Every external cohort was also split into two groups in terms of their median RS.

To evaluate the performance of the model, we used TCGA-PAAD as the internal validation and GSE57495 and CPTAC as the external validations by calculating the area under the curves (AUC) of the receiver operating characteristic (ROC) curve, analyzing the Kaplan–Meier curves (K-M curves), and assessing the Harrell’s concordance index (C-index). By analyzing the K-M curves in different clinical characteristics, TCGA-PAAD was employed in testing the adaptability of the model.

### Establishment and evaluation of nomograms

In TCGA-PAAD, univariate and multivariate Cox regressions were used to select the independent prognostic factors. In light of these factors, we built the nomograms, which included age, sex, grade, distant metastasis, lymph node metastasis, and risk score. The ROC and decision curve analysis (DCA) were applied to estimate the nomograms.

### Protein–protein interaction and gene set enrichment analysis

The interaction network of the protein encoded by the genes that constituted the formula of the risk score was analyzed by the STRING database (https://string-db.org/), with an interaction score >0.7. The interaction map was drawn by the Cytoscape package (version 3.9.1).

The Gene set enrichment analysis (GSEA) was performed to discover the latent enriched pathways in the low- and high-risk groups in TCGA-PAAD based on the Kyoto Encyclopedia of Genes and Genomes (KEGG), Gene Ontology (GO), and HALLMARK, in accordance with the methods by the R packages “GSEA” and “FGSEA” (Subramanian et al., 2005; Korotkevich et al., 2021). The criteria were set as |NES| > 1, false discovery rate (FDR, *p* adjusted) < 0.25, and *p* < 0.05.

### Immune cell and immune-related signature

To evaluate the infiltration of immune cells in clusters, we utilized the multiple R package algorithms, which included “CIBERSORT,” “quanTIseq,” “TIMER,” “MCPcounter,” “EPIC,” and “ssGSEA,” and the immune cells included T cells, CD8^+^ T cells, B cells, cytotoxic lymphocytes (CTLs), endothelial cells, fibroblasts, monocytic lineage, myeloid dendritic cells (mDCs), neutrophils, natural killer (NK) cells, and other immune cells.

To excavate the potential novel immunotherapy target, the Wilcoxon signed-rank test was introduced to explore the well-known immune-related genes differently expressed between high- and low-risk groups, and the STRING database was used to find the relevant pathways about these genes (Thorsson et al., 2019).

### Genomic profile

The mutation data were downloaded from the TCGA database. The “maftools” package was used to visualize the mutation data of the variant type, significantly mutated genes, substitution mutation, Catalogue of Somatic Mutations in Cancer (COSMIC) signature, and interaction of mutations in the high- and low-risk groups (TCGA-PAAD) (Mayakonda et al., 2018). The oncogenic pathways and alteration of copy number variations (CNVs) were also analyzed by R “maftools”.

### Single-cell RNA sequencing characteristics

Cell clustering was achieved by the principal component analysis (PCA) and R “Seurat.” “TSNE” was used to visualize the clustering state, and the clusters were marked based on ductal cells (*KRT19*, *KRT7*, *TSPAN8*, and *SLPI*), stellate cells (*RGS5*, *ACTA2*, *PDGFRB*, and *ADIRF*), fibroblasts (*LUM*, *DCN*, *COL1A1*, and *C1R*), T cells (*CD3D*, *CD3E*, *CD4*, *CD8A*, *CD8B*, *CD2*, and *CXCR4*), and myeloid cells (*AIF1*, *CD14*, *CD68*, *LILRA4*, and *CXCR3*) (Peng et al., 2019). The differentially expressed genes between the high- and low-risk groups were identified by “Seurat” with the “FindMarkers” function. The enriched pathways in the two RS groups were determined by GSEA and GSVA. The setting for GSEA was both FDR and *p* values <0.05, while for GSVA, it was the correlation coefficient >1.

### Chemotherapy and immunotherapy response

On the basis of clinical recommendations, the regimens, which included gemcitabine plus paclitaxel and FOLFIRINOX (5-fluorouracil, oxaliplatin, irinotecan, and leucovorin), were selected as the standard chemotherapy for PC patients. *KRAS G12C* inhibitor is a new drug targeted at *KRAS* mutation, which is one of the most common alterations in PC patients. To predict the sensitivity of these drugs, the Genomics of Drug Sensibility in Cancer Database (GDSC, https://www.cancerrxgene.org/) was applied to estimate the half-maximal inhibitory concentration (IC50) of the samples in the low- and high-risk groups (TCGA-PAAD).

To clarify the potential value of our model with respect to immunotherapy, we analyzed T-cell inflamed gene expression profile (GEP), cytotoxic activity (CYT), and the Tumor Immune Dysfunction and Exclusion (TIDE) and obtained relevant information from the immunotherapeutic cohort (IMvigor210) treated with the anti-PD-L1 agent atezolizumab and calculated the K-M curves of TCGA-PAAD (Cristescu et al., 2018; Jiang et al., 2018; Mariathasan et al., 2018). Based on the response evaluation criteria in solid tumors (RECIST), immunotherapy treatment in patients was identified as complete or partial response (CR/PR) and stable disease (SD) or progressive disease (PD). The relationship of the risk groups with the efficacy of treatment was analyzed by using the Fisher’s test.

### Protein and mRNA expression

The immunohistochemical data of normal pancreatic and cancer tissues were acquired from the Human Protein Atlas (HPA, https://www.proteinatlas.org/) to evaluate the protein expressions of MET, FERMT1, MMP3, and CARD9. Based on the mRNA expression from the TCGA database, HPA analyzed the survival rate of approximately five genes in PC patients.

### Cell lines and culture

Human PC cell lines, MIA PaCa-2, and the normal pancreatic cell line, hTRET-HPNE, were cultured in Dulbecco's modified Eagle medium (DMEM) (VivaCell, Germany), with 10% FBS (Evergreen, China) and 1% penicillin G (100 U/mL) (Beyotime Biotechnology, China). In addition, other PC cell lines, CAPAN-1 and CFPAC-1, were incubated in Iscove’s modified Dulbecco’s medium (IMDM) (VivaCell, Germany), supplied with 20% FEB, and 10% FBS (Evergreen, China) and 1% penicillin G (100 U/mL) (Beyotime Biotechnology, China). The cells were cultivated at 37°C with 5% CO_2_ and were collected at 80% confluence.

### Quantitative reverse transcription PCR

The cells were harvested, and RNA was extracted by RNAiso Plus (Takara, Kusatsu, Japan). Following the instructions, RNA was reversely transcript into cDNA, using a PrimeScript™ RT reagent Kit with gDNA Eraser (Takara, Kusatsu, Japan). PCR was performed on Bio-Rad CFX (Bio-Rad, United States) with TB Green^®^ Premix EX Taq™ II (Tli RNase H Plus) (Takara, Kusatsu, Japan). GAPDH was considered a housekeeping gene. The expression of five genes was analyzed using the 2^−ΔΔCT^ [∆CT = CT (target gene), CT (housekeeping gene), ∆∆CT = ∆CT (cancer cell line), and ∆CT (normal cell line)]. The primer sequences are listed in Table 1. We compared every gene expression between each PC cell line and normal pancreatic cell line. All experiments were repeated thrice.

**TABLE 1:** Primer sequences of genes.

	Forward primer (5′-3′)	Reverse primer (5′-3′)
CARD9	ATG​TCG​GAC​TAC​GAG​AAC​GAT	TGA​TGC​GTG​AGG​GGT​CGA​T
IL20RB	AGG​CCC​AGA​CAT​TCG​TGA​AG	CGA​CCA​CAA​GGA​TCA​GCA​TGA
MMP3	AGT​CTT​CCA​ATC​CTA​CTG​TTG​CT	TCC​CCG​TCA​CCT​CCA​ATC​C
MET	CTA​GAC​ACA​TTT​CAA​TTG​GT	TGT​TGC​AGG​GAA​GGA​GTG​GT
FERMT1	GCG​TTG​ACC​ATC​CCA​ATG​AAG	ACC​AAA​GAG​CAA​AGT​CTG​ACC
GAPDH	GAA​ATC​CCA​TCA​CCA​TCT​TCC​AGG	GAG​CCC​CAG​CCT​TCT​CCA​TG

### Statistical analysis

The statistical analysis and relevant figure drawings were performed by R (version 4.1.2). The comparison of continuous variables in the two groups was made by the Wilcoxon test and *t*-test. The significance of survival was calculated by K-M curves and Cox regression. The correlation analysis between groups was analyzed by the Fisher’s test, and the tables were drawn by using EXCEL. LASSO and univariate and multivariate Cox regressions were used for the analysis of prognosis-related genes. The univariate and multivariate Cox regressions were applied to assess the relationship between prognosis and clinical features and the risk score. *p* < 0.05 was considered statistically significant.

## Results

### Risk model

#### Identification of differentially expressed genes and building a prognostic risk model

**FIGURE 2:**
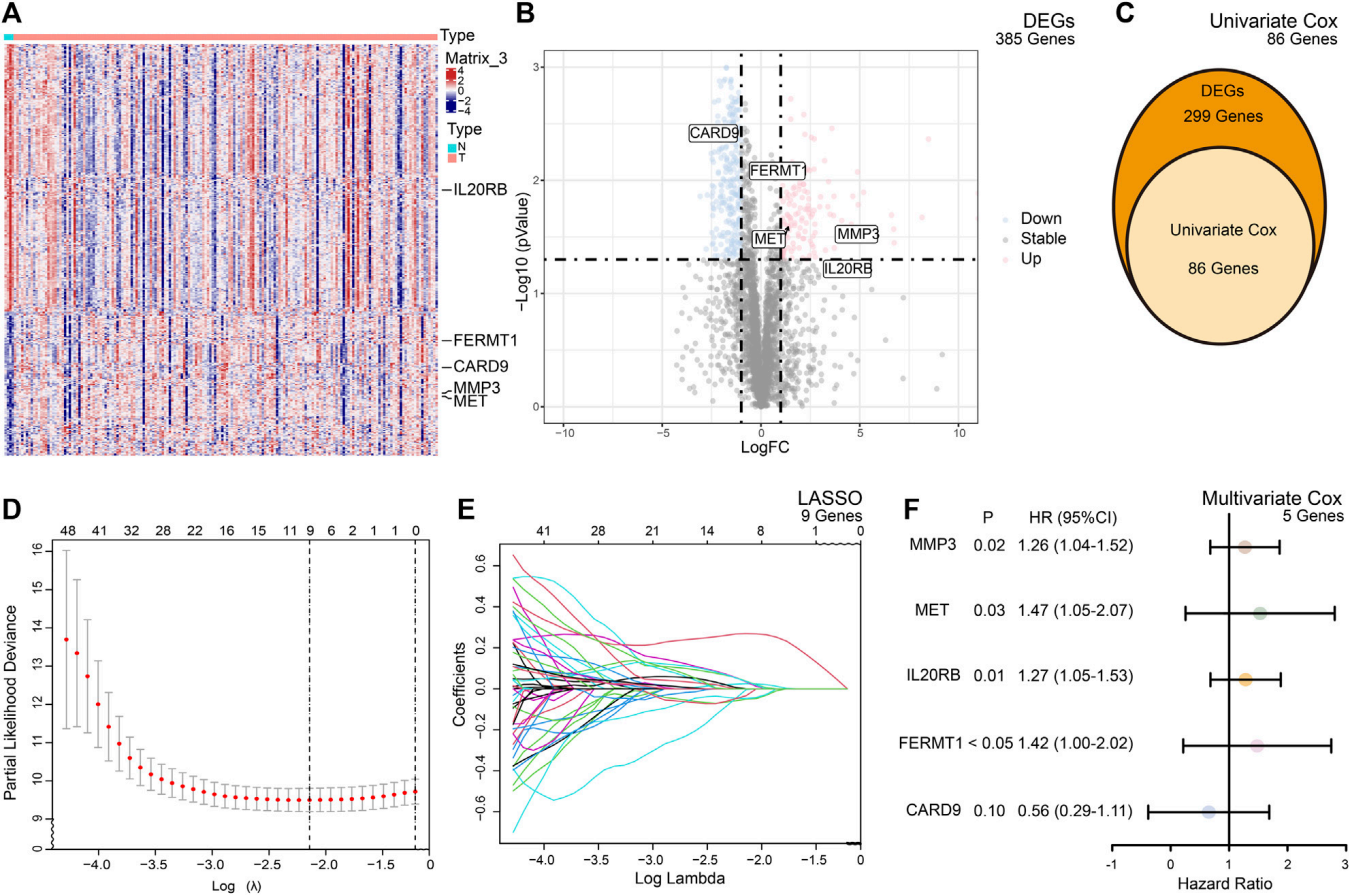
Summary of the differentially expressed genes (DEGs) and model building. **(A)** Heatmap of the expression of the DEGs between the normal and cancer tissues of PC. **(B)** Volcano plot of the expression of the DEGs between the normal and cancer tissues of PC. **(C)** Based on DEGs, univariate Cox analysis selected 86 candidate genes for risk model building. **(D)** Cross validation indicated minimum criteria for tuning parameter selection (*λ*) in the LASSO is nine. **(E)** LASSO coefficient profiles of the TME-related genes. **(F)** Multivariate Cox analysis of selected genes.

The TME participates in the process of PC. To study the molecular signature of the TME in PC, we retrieved 4,709 TME-related genes from the ImmPort Portal and MsigDB in total and finally identified 385 genes which were differentially expressed between normal and cancer tissues (Figures 2A, B).

To predict the outcomes of PC patients, we built a prognostic risk model. After univariate and multivariate Cox and LASSO analyses, five genes, namely, *FERMT1*, *CARD9*, *IL20RB*, *MET*, and *MMP3*, met the criteria and constituted the formula of the risk model (Figures 2C, D). The formula was formed by the sum of the products of the expression of the gene and its coefficient RS=0.35×FERMT1−0.57×CARD9+0.24×IL20RB+0.39×MET+0.23×MMP3 .

#### Internal and external validation of risk model

**FIGURE 3:**
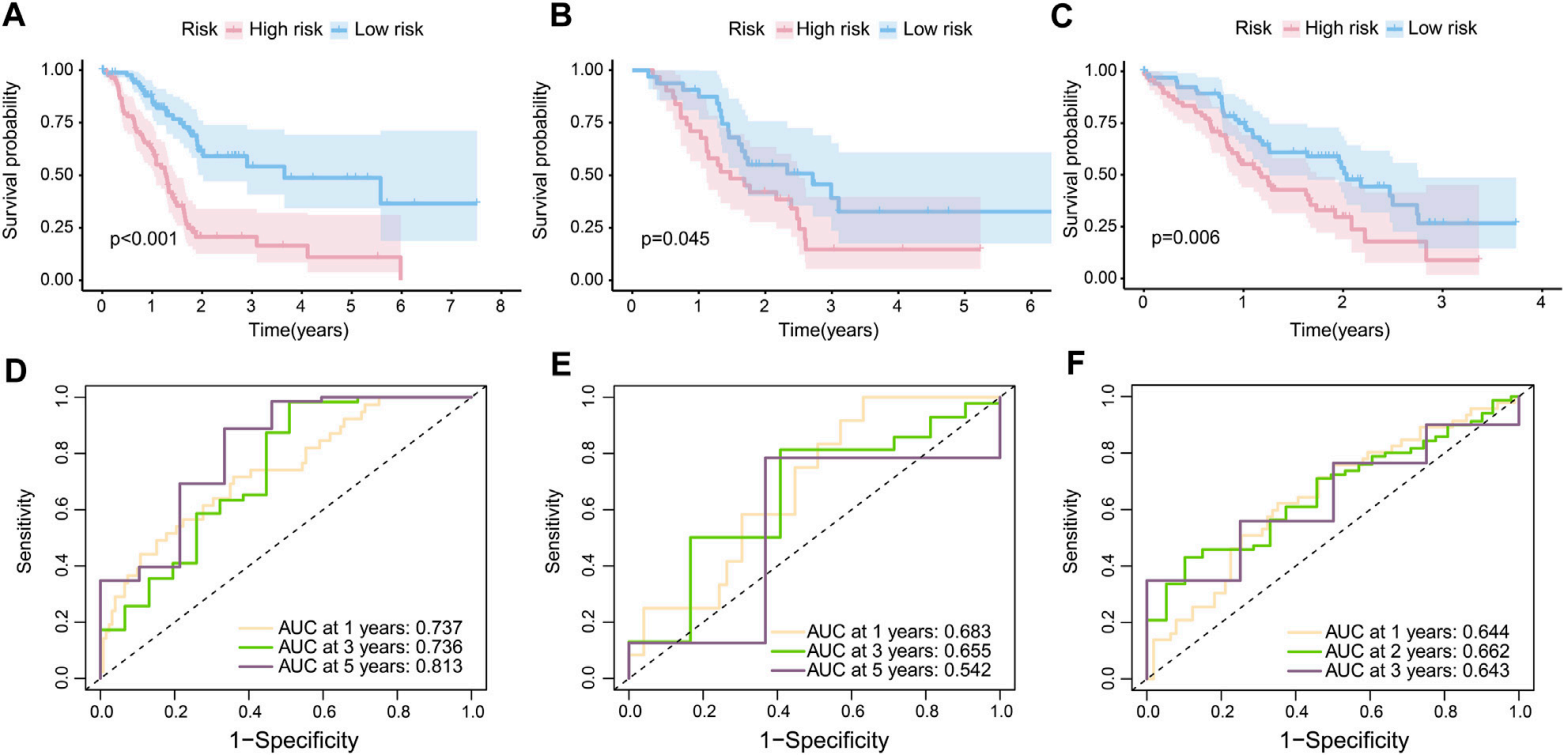
Internal and external validation of the risk model. **(A)** K-M curves of the overall survival in TCGA-PAAD (*p* < 0.001). **(B)** K-M curves of the overall survival in GSE57495 (*p* < 0.05). **(C)** K-M curves of the overall survival in CPTAC (*p* < 0.05). **(D)** 1-year, 3-year, and 5-year ROC curves of the risk score in the TCGA-PAAD cohort; **(E)** 1-year, 3-year, and 5-year ROC curves of the risk score in the GSE57495 cohort; and **(F)** 1-year, 2-year, and 3-year ROC curves of the risk score in the CPTAC cohort.

In accordance with the RS, 86 samples were assigned to the high-risk group, with 91 samples being assigned to the low-risk group in TCGA-PAAD. In GSE57495, 31 patients were assigned to the high-risk group and 32 patients to the low-risk group. In CPTAC, 67 patients were assigned to the low-risk group and the rest were labeled as high risk. The K-M curves confirmed that patients with high-risk scores were prone to having a poor prognosis in TCGA-PAAD (*p* < 0.001) (Figure 3A), GSE57495 (*p* < 0.05) (Figure 3B), and CPTAC (*p* < 0.01) (Figure 3C). Additionally, the ROC curve indicated that our prognostic risk model was widely flexible, with AUCs at 1 year, 3 years, and 5 years of 0.737, 0.736, and 0.813, respectively, in the TCGA-PAAD cohort (Figure 3D). The AUCs at 1 year, 3 years, and 5 years were 0.683, 0.655, and 0.542 in cohort GSE57495, respectively (Figure 3E). The AUCs at 1 year, 2 years, and 3 years were 0.644, 0.662, and 0.643 in cohort CPTAC, respectively (Figure 3F).

#### Evaluation of risk model

**FIGURE 4:**
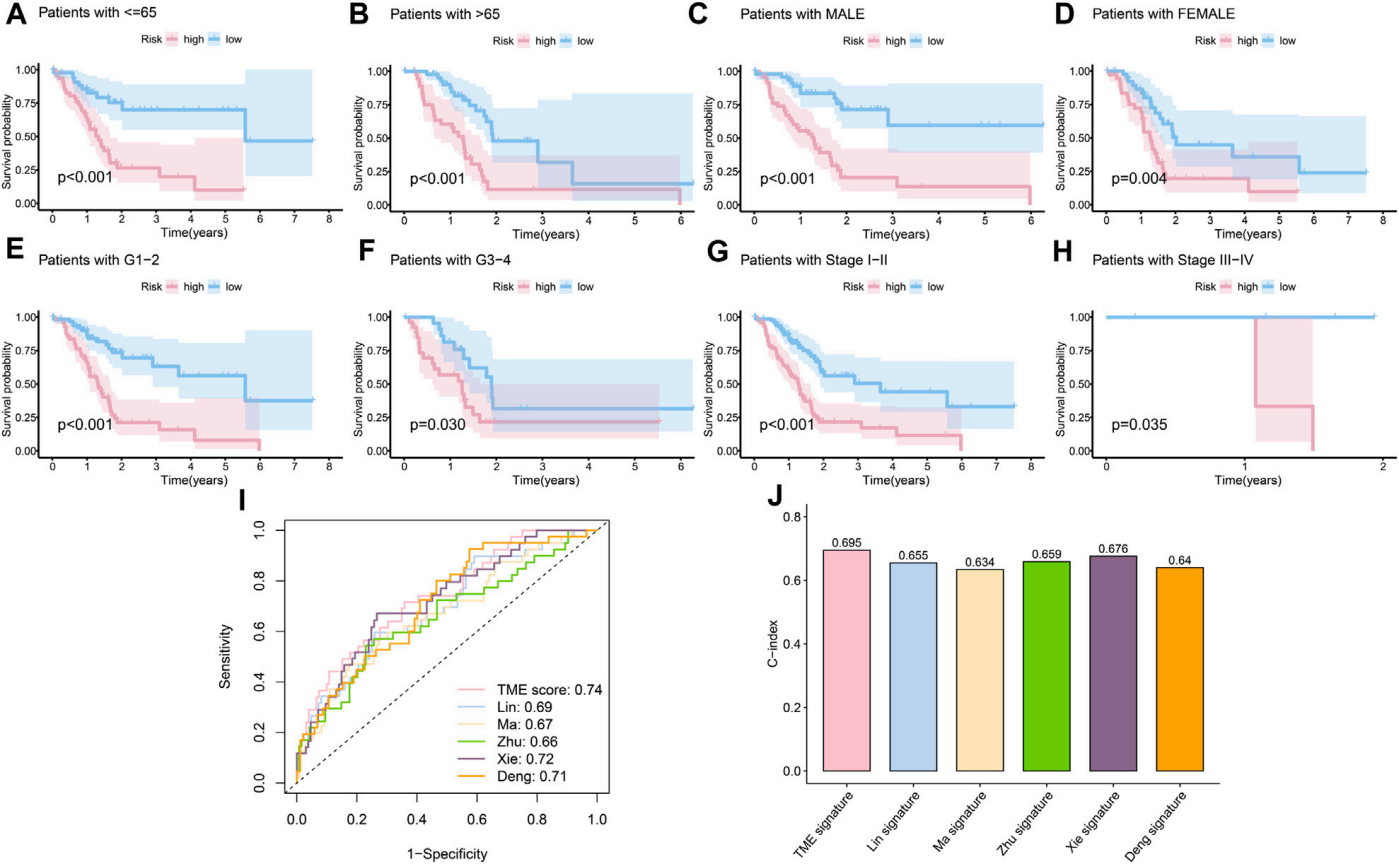
Evaluation of the risk model. **(A)** K-M curves of the survival probability in patients under 65 years old (*p* < 0.001). **(B)** K-M curves of the survival probability in patients over 65 years old (*p* < 0.001). **(C)** K-M curves of the survival probability in male patients (*p* < 0.001). **(D)** K-M curves of the survival probability in female patients (*p* < 0.01). **(E)** K-M curves of the survival probability in patients with grades 1–2 disease (*p* < 0.001). **(F)** K-M curves of the survival probability in patients with grades 3–4 disease (*p* < 0.05). **(G)** K-M curves of the survival probability in patients with stage 1–2 disease (*p* < 0.001). **(H)** K-M curves of the survival probability in patients with stages 3–4 disease (*p* < 0.05). **(I)** 1-year ROC curves of the risk score in Lin et al. (2021), Ma et al. (2021), Zhu et al. (2021), Xie et al. 2022, Deng et al. (2022), and our models. **(J)** C-index of different models.

The clinical features were used to test the applicability of the model. Based on the different clinical indices, such as age, sex, grade, and stage, the patients were separated into different subgroups. The K-M curves demonstrated that there were notable prognostic differences between the low- and high-risk groups in ages ≤65 years (*p* < 0.001) (Figure 4A); ages >65 years (*p* < 0.001) (Figure 4B); male sex (*p* < 0.001) (Figure 4C); female sex (*p* < 0.01) (Figure 4D); grades 1–2 (*p* < 0.001) (Figure 4E); grades 3–4 (*p* < 0.05) (Figure 4F); stages 1–2 (*p* < 0.001) (Figure 4G); and stages 3–4 (*p* < 0.05) (Figure 4H). The univariate Cox regression indicated that the prognosis was related to age (*p* < 0.05), grade (*p* < 0.05), and RS (*p* < 0.001), while the multivariate Cox regression showed that only age (*p* < 0.05) and RS (*p* < 0.001) were independent prognostic factors (Table 2). In general, RS is a reliable independent index for prognosis.

**TABLE 2:** Relationship between clinical characteristics and prognosis via univariate and multivariate Cox regression^a^.

	Univariate Cox analysis			Multivariate Cox analysis		
	HR	95% CI	*p*	HR	95% CI	*p*
Age	1.03	(1.01–1.05)	0.01	1.03	(1.00–1.05)	0.03
Gender	0.90	(0.59–1.37)	0.61			
Grade	1.38	(1.02–1.86)	0.04	1.28	(0.93–1.75)	0.13
Stage	1.42	(0.98–2.07)	0.06			
Risk score	1.16	(1.09–1.23)	5.75e-07	1.16	(1.09–1.22)	1.04e-06

^a^ One cancer sample was retrieved after chemotherapy, but the information of clinical characteristics of all patients was collected before treatments.

Next, considering the lack of data on long survival in PC, we compared our risk model with published models on ROC at 1 year, and our model achieved the largest AUC in the data set TCGA-PAAD (Figure 4I), achieved the fourth largest AUC in CPTAC (Supplementary Figure S1A), and the second in GEO57495 (Supplementary Figure S1B). The c-index of our model is 0.70, which is higher than the c-index values of Lin et al. (2021) (0.66); Ma et al. (2021) (0.63); Zhu et al. (2021) (0.66); Xie et al. 2022 (0.68); and Deng et al. (2022) (0.64) models (Figure 4J). The c-index values of our model based on CPTAC and GEO57495 were 0.595 (Supplementary Figure S1C) and 0.596 (Supplementary Figure S1D), respectively. The evidence verified that our risk model was superior to those of the other methods in terms of prognosis prediction.

#### Establishment of nomogram and DCA

**FIGURE 5:**
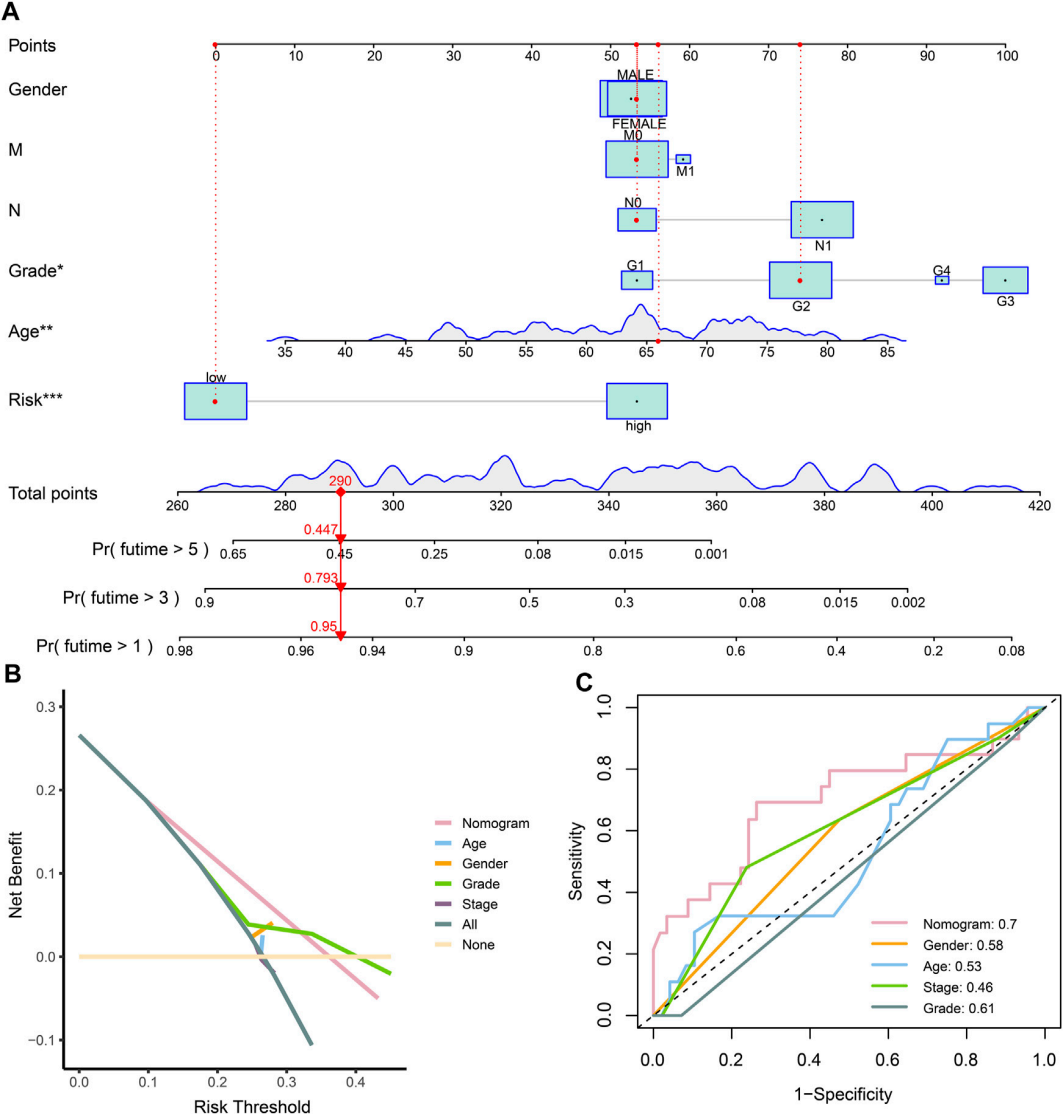
Establishment and evaluation of the nomogram. **(A)** Nomogram of clinical characteristics and risk score. **(B)** DCA of clinical characteristics and the nomogram. **(C)** ROC of clinical characteristics and the nomogram.

Considering the prognostic value of the risk score and clinical characteristics, a nomogram was established to comprehensively predict the outcome. The 1-, 3-, and 5-year survival rates could be calculated by adding the points obtained using age, sex, distant metastasis, lymph node metastasis, grade, and risk score. Moreover, the model could help decision-makers manage patients reasonably and foresee the prognosis of patients (Figure 5A). The DCA and ROC curve showed that the prediction of prognosis benefited more from the nomogram than it did from the assessment with a single clinical index (Figures 5B, C).

### Molecule and immune features

#### Molecular characteristics of high- and low-risk groups

**FIGURE 6:**
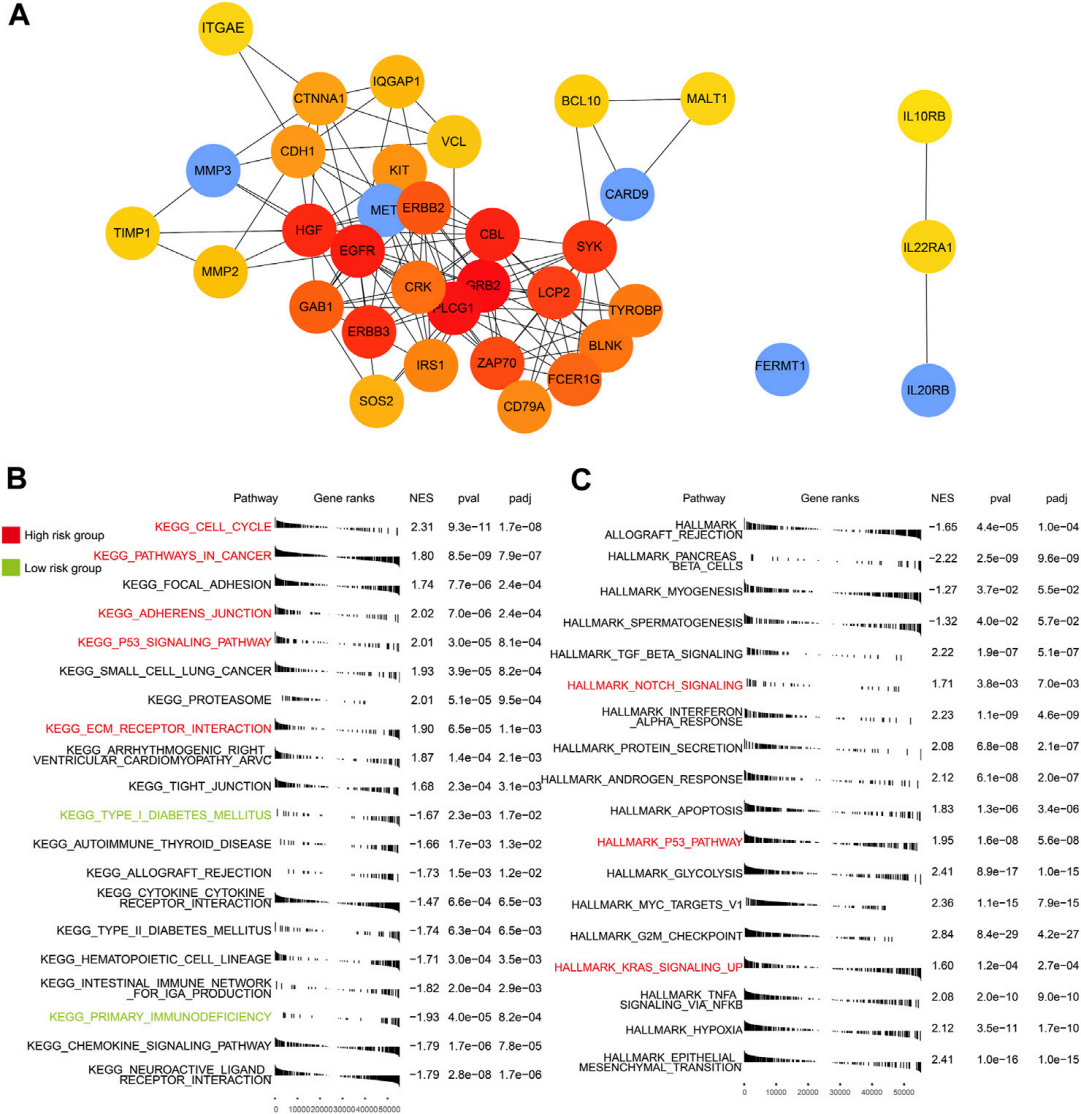
PPI of five genes and GSEA of high- and low-risk groups. **(A)** The interaction of proteins focused on the five genes. **(B)** Enriched KEGG pathways in the TCGA-PAAD (*p* < 0.05). **(C)** Enriched HALLMARK pathways in the TCGA-PAAD (*p* < 0.05). Key pathways marked in red are mainly enriched in the high-risk group and those in green are in the low-risk group.

Based on protein–protein interaction (PPI) in the STRING database, 35 molecular targets were labeled to be associated with the four genes (FREMT1 being the exception) that were included in the risk model (Figure 6A).

We identified a few critical KEGG (Figure 6B), GO (Supplementary Figure S2), and HALLMARK (Figure 6C) pathways in each subgroup. In the high-risk group, multiple cancer-related and ECM relative pathways were upregulated, which included pathways in cancer (*p* < 0.05), the cell cycle (*p* < 0.05), *KRAS* signaling up (*p* < 0.05), adherens junction (*p* < 0.05), ECM receptor interaction (*p* < 0.05), and notch signaling (*p* < 0.05). Nevertheless, the immune pathways, which included primary immunodeficiency (*p* < 0.05) and adaptive immune response (*p* < 0.05), and the endocrine and metabolic related pathways, such as type I diabetes mellitus (*p* < 0.05) and type II diabetes mellitus groups (*p* < 0.05), were mainly enriched in the low-risk group. The pathway related to cancer and the lack of involvement of immunity may explain the poor outcomes of patients in the high-risk group.

#### Immune cell infiltration and immune signature in high- and low-risk groups

**FIGURE 7:**
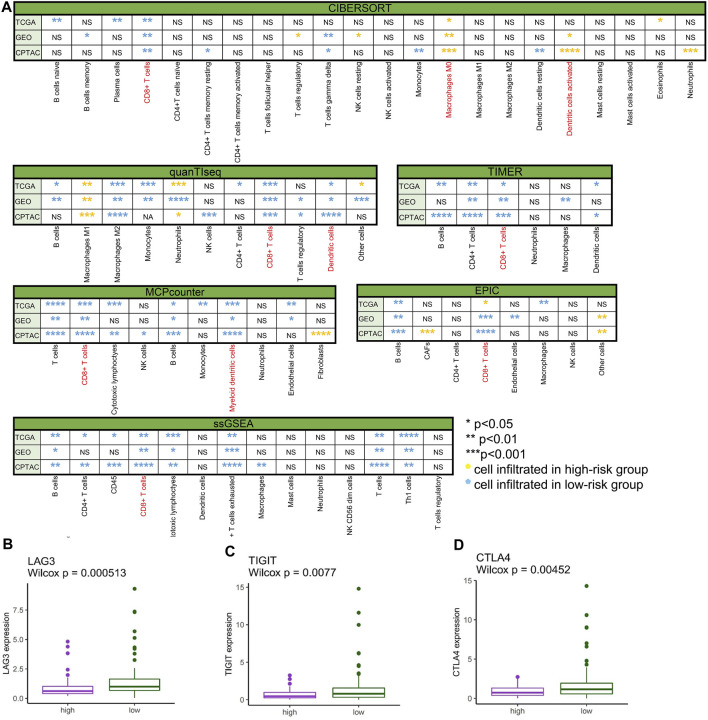
Immune cell infiltration and gene expression. **(A)** Comparison of immune cell infiltration between the low- and high-risk groups in TCGA-PAAD, GSE57495, and CPTAC analyzed by CIBERSORT, quanTIseq, TIMER, MCPcounter, EPIC, and ssGSEA (*p < 0.05, **p < 0.01, ***p < 0.001, ****p < 0.0001, “ns” no significance, yellow* immune cell mainly infiltrated in the high-risk group, and blue* immune cell mainly infiltrated in the low-risk group). **(B)** LAG3 differently expressed in high- and low-risk groups in TCGA-PAAD (*p* < 0.001). **(C)** TIGIT differently expressed in high- and low-risk groups in TCGA-PAAD (*p* < 0.01). **(D)** CTLA4 differently expressed in high- and low-risk groups in TCGA-PAAD (*p* < 0.01).

To comprehensively investigate the distribution of immunocytes, we applied the multiple algorithms in every cohort. Generally, immune cells differently infiltrated in the three cohorts. We noticed that T cells, CD8^+^ T cells, B cells, and CTLs were inclined to cluster in the low-risk group in the three cohorts based on different algorithms (Figure 7A) (*p* < 0.05). In contrast to the low-risk group, the patients in the high-risk group exhibited lower infiltration of immune cells.

To spot the latent immune therapy target, we analyzed the expression of immune genes in different groups in TCGA-PAAD. The results showed that *LAG3* (Figure 7B), *TIGIT* (Figure 7C), and *CTLA-4* (Figure 7D) were expressed more in the low-risk group than in the high-risk group (*p* < 0.01). Preclinical and clinical studies have shown that these genes are prospective immunotherapy targets (Chen et al., 2022).

### Genomic patterns

#### Key mutation signatures

**FIGURE 8:**
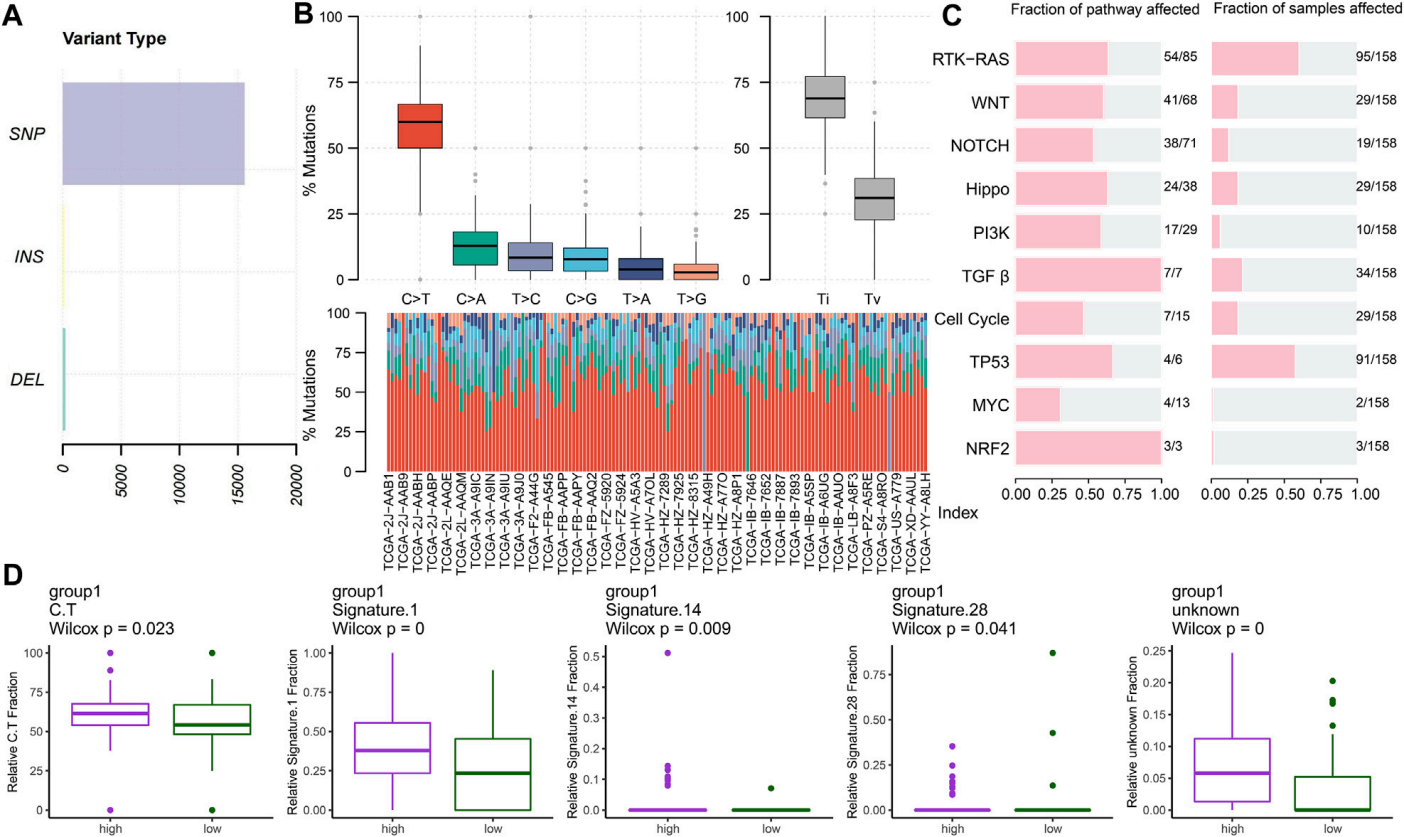
Key mutation signatures. **(A)** Summary of the variant type in the TCGA-PAAD cohort. **(B)** Possible substitution types in TCGA-PAAD. **(C)** Mutation condition about oncogenic pathways in TCGA-PAAD. **(D)** Different expressions of the COSMIC mutational signatures in TCGA-PAAD (*p* < 0.05).

We analyzed the mutational spectrum of TCGA-PAAD patients in the high- and low-risk groups. SNP was the most common variant type (Figure 8A). In total, 23,333 substitutions occurred in 149 samples, with the range from 0 to 2,371. In addition, the C>T substitution was the most distinct one between the high- and low-risk groups (*p* < 0.05) (Figure 8B). In light of the COSMIC signatures that were generated by decomposing the mutation profile, the contribution of signatures 1, 14, and 28 exhibited significant differences in the two subgroups (*p* < 0.05) (Figure 8D). C>T mutations most likely arise from the T:G mismatches generated from the deamination of 5′-methylcytosine because of non–prior repair during DNA replication. In addition, this is the characteristic of signature 1 which works as a cell division/mitotic clock in most cancers (Alexandrov et al., 2015).

Understanding the mechanisms of somatically altered signaling pathways in cancer is critical to develop new therapeutic approaches (Sanchez-Vega et al., 2018). By “maftools,” we found that the RTK-RAS and TP53 pathways were the top two signaling pathways with the frequency of alterations, representing 60.13% and 57.59%, respectively (Figure 8C).

#### Significantly mutated genes

**FIGURE 9:**
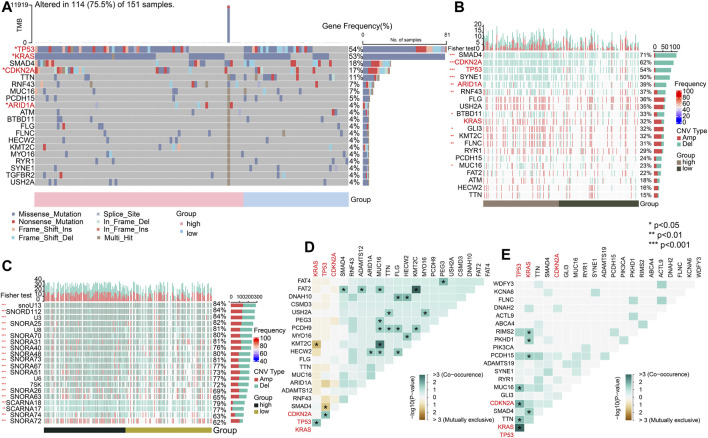
Significant mutated genes. **(A)** Top 20 mutated genes in high- and low-risk groups of the TCGA-PAAD cohort. **(B)** CNV condition of top 20 mutated genes. **(C)** Top 20 mutated genes by CNV. **(D)** Interaction of gene mutations in the high-risk group. **(E)** Interaction of gene mutations in the low-risk group. (*p < 0.05, **p < 0.01, ***p < 0.001, and ****p < 0.0001).

To explore the genomic alterations between the high- and low-risk groups, we identified mutated genes and investigated the CNVs. As previously reported, the top mutated genes, that is, *TP53*, *KRAS*, and *CDKN2A*, showed the most single-nucleotide variations (SNVs) in the high-risk group in PC (Figure 9A) (*p* < 0.05). The pattern that mutation appeared in the high-risk group with none of it appearing in the low-risk group only showed for *ARID1A* (*p* < 0.05). To gain more mutational perspectives, we analyzed the CNVs of the mentioned top 20 genes and obtained similar results with the frequency of mutation of *TP53*, *CDKN2A*, and *ARID1A* (*p* < 0.05), while the CNVs of *KRAS* showed no significant difference in the high- and low-risk groups (*p* > 0.05) (Figure 9B). Next, the striking alterations of CNVs were investigated. In addition, the mutations of CNVs were mainly snoRNAs, the job of which was uncertain in PC (*p* < 0.05) (Figure 9C) (Williams and Farzaneh, 2012). These results indicate the candidates of CNVs for intimate relationships with PC. Using the maftools, the interaction of SMGs was described. In the high-risk group, it is notable that *TP53* chiefly co-mutated with *KRAS* (*p* < 0.05) (Figure 9D), whereas in the low-risk group, it mostly co-mutated with *CDKN2A* (*p* < 0.05) (Figure 9E).

#### Single-cell RNA sequencing characteristics

**FIGURE 10:**
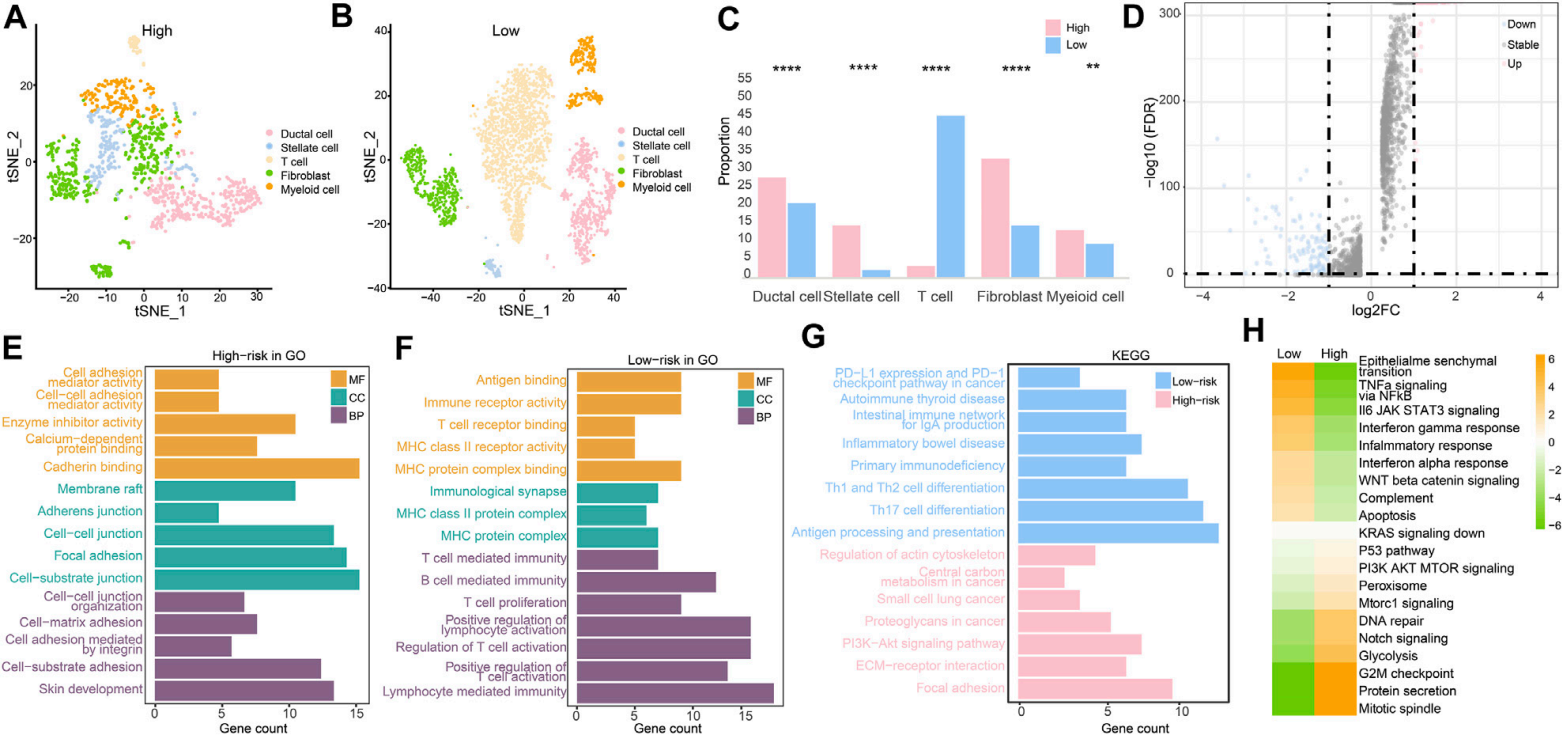
Single-cell RNA sequencing analysis. **(A)** Cell clustering in the high-risk group. **(B)** Cell clustering in the low-risk group. **(C)** Different analysis of cell clustering in high- and low-risk groups by Fisher’s test. **(D)** Differentially expressed genes in high- and low-risk groups. **(E)** Enriched pathways from GO in the high-risk group. **(F)** Enriched pathways from GO in the low-risk group. **(G)** Enriched pathways from KEGG in two RS groups. **(H)** Enriched pathways by GSVA in two RS groups. (*p < 0.05, **p < 0.01, ***p < 0.001, and ****p < 0.0001).

After clustering and annotation of clusters, we noticed the distributions of cancer cells, and fibroblasts in the high-risk groups were apparently higher than those in the low-risk group (*p* < 0.0001), while myeloid cells were slightly highly clustered in the high-risk group than they were in the low-risk group (*p* < 0.01). However, T cells were merely expressed in the high-risk group, while they mostly clustered in the low-risk group (*p* < 0.0001) (Figures 10A–C). These evidence revealed that immune microenvironments in the two RS groups were diverse. Based on the differentially expressed genes (*p* < 0.05) (Figure 10D), enriched pathways from GO, KEGG, and HALLMARK were analyzed (Figures 10E–H). Similar to the results of the enriched pathways in the analysis of transcriptome, the immune-related pathways were chiefly enriched in the low-risk group, as cancer- and adhesion-related pathways were largely boosted in the high-risk group (*p* < 0.05).

#### Chemotherapy and immunotherapy response

**FIGURE 11:**
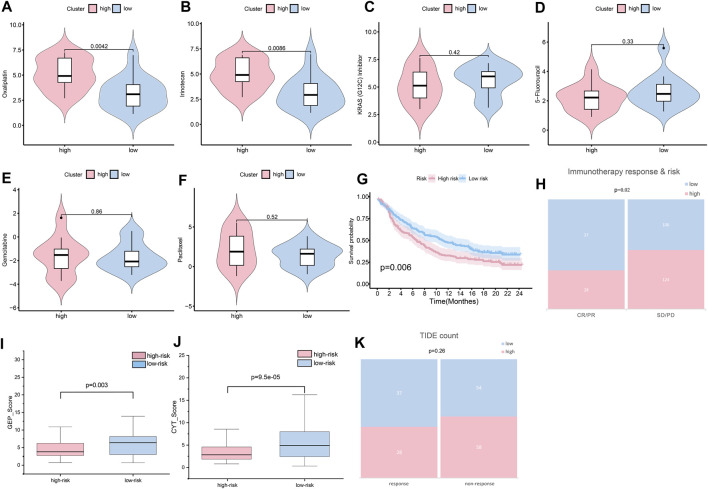
Sensitivity of patients to treatments. **(A)** Oxaliplatin sensitivity in the high- and low-risk groups (*p* < 0.01). **(B)** Irinotecan sensitivity in the high- and low-risk groups (*p* < 0.01). **(C)** KRAS (G12C) sensitivity in the high- and low-risk groups (*p* > 0.05). **(D)** 5-fluorouracil sensitivity in the high- and low-risk groups (*p* > 0.05). **(E)** Gemcitabine sensitivity in the high- and low-risk groups (*p* > 0.05). **(F)** Paclitaxel sensitivity in the high- and low-risk groups (*p* > 0.05). **(G)** Assuming K-M curves of the survival probability in patients in different groups after immunotherapy (*p* < 0.01). **(H)** Assuming response to immunotherapy in different groups (*p* < 0.05). **(I)** GEP score in the high- and low-risk groups (*p* < 0.05). **(J)** CYT score in the high- and low-risk groups (*p* < 0.001). **(K)** TIDE count in the high- and low-risk groups (*p* > 0.05).

In validating chemotherapy sensitivity of different groups, based on the drug response data, using a panel of 29 PC cells, the high-risk cell lines were more resistant to oxaliplatin and irinotecan (the typical chemotherapy drug for PC) than the low-risk cell lines (*p* < 0.01) (Figures 11A, B). The IC50 of *KRAS* (G12C) inhibitor was higher in the low-risk group than it was in the high-risk group; however, the difference was not significant (Figure 10C). In addition, other drugs, such as 5-fluorouracil, gemcitabine, and paclitaxel, showed little distinction between the low- and high-risk groups (*p* > 0.05) (Figures 11D–F).

Immunotherapy is widely and successfully used in the treatment of many cancers. To determine the potential response to immunotherapy in PC, an anti-PD-1 cohort IMvigor210 was used in our analysis. Patients labeled for high risk benefitted little from the treatment with atezolizumab, while low-risk patients obtained better outcomes (*p <* 0.01) (Figure 11G). After immunotherapy, patients with lower risk scores were more likely to have a complete response or partial response (CR/PR) (*p <* 0.05) (Figure 11H). As reported, GEP and CYT are promising therapeutic indexes for PD-1 blockade. Our results confirmed that the low-risk group was prone to higher GEP (Figure 11I) and CYT (Figure 11J) scores (*p* < 0.01). The low-risk group was more likely to achieve a response after immune treatment, although the difference was not significant (Figure 11K). Generally, the risk score could provide clinicians with a method for identifying beneficial treatment for PC patients.

#### Protein expression of genes in pancreatic tissues

**FIGURE 12:**
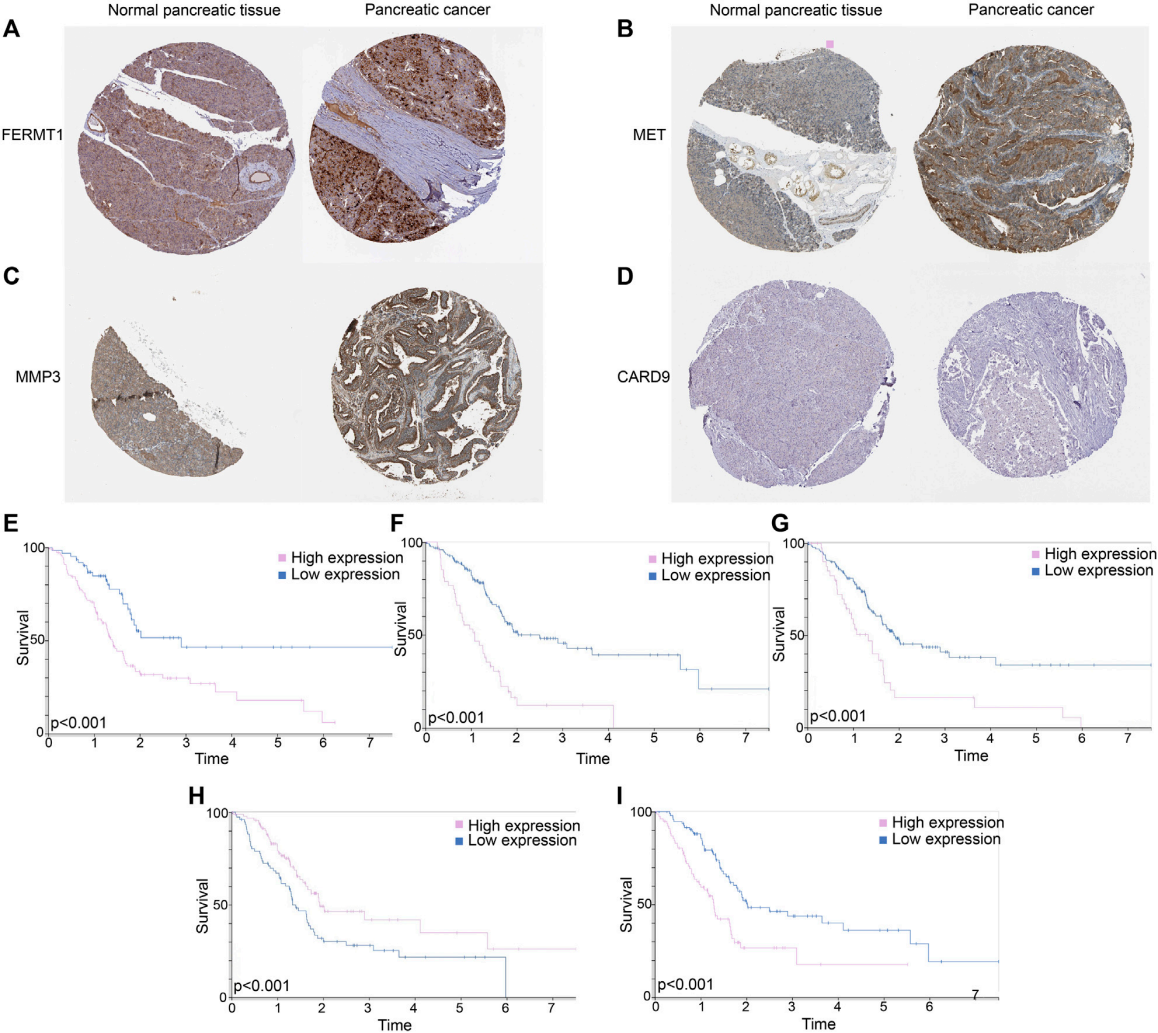
**(A)** Immunohistochemical data of FERMT1 in normal and tumor tissues in PC. **(B)** Immunohistochemical data of MET in normal and tumor tissues in PC. **(C)** Immunohistochemical data of MMP3 in normal and tumor tissues in PC. **(D)** Immunohistochemical data of CARD9 in normal and tumor tissues in PC. **(E)** K-M curves of survival probability in patients whose expressions of FERMT1 were different. **(F)** K-M curves of survival probability in patients whose expressions of MET were different. **(G)** K-M curves of survival probability in patients whose expressions of MMP3 were different. **(H)** K-M curves of survival probability in patients whose expressions of CARD9 were different. **(I)** K-M curves of survival probability in patients whose expressions of IL20RB were different.

FERMT1 (Figure 12A), MET (Figure 12B), and MMP3 (Figure 12C) overexpressed in PC tissues, while the expressions of CARD9 (Figure 12D) were not outstanding in both normal and cancer tissues in PC. According to the K-M analyses from HPA, all five genes were prognosis indexes for PC patients. Overexpression of *FERMT1* (Figure 12E), *MET* (Figure 12F), *MMP3* (Figure 12G), and *IL20RB* (Figure 12I) showed worse survival for PC. However, better prognostic tendencies showed up in PC patients who exhibited high expressions of *CARD9* (Figure 12H). These results reconfirmed our model.

#### MRNA expression in PC samples and cells

**FIGURE 13:**
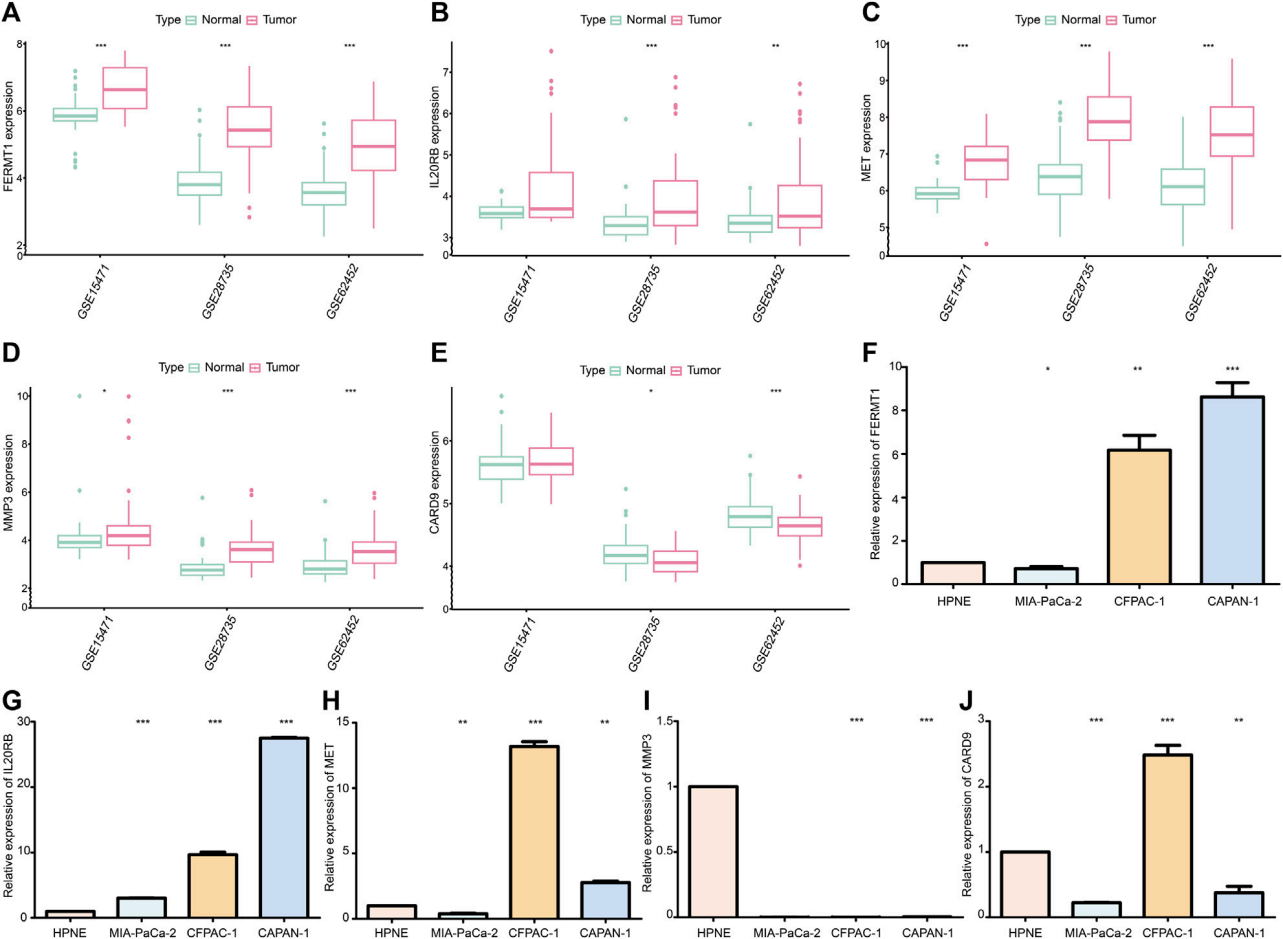
**(A)** Difference in mRNA expression of *FERMT1* between normal and cancer tissues in data sets GSE15471, GSE28735, and GSE62452. **(B)** Difference in mRNA expression of *IL20RB* between normal and cancer tissues in data sets GSE15471, GSE28735, and GSE62452. **(C)** Difference in mRNA expression of *MET* between normal and cancer tissues in data sets GSE15471, GSE28735, and GSE62452. **(D)** Difference in mRNA expression of *MMP3* between normal and cancer tissues in data sets GSE15471, GSE28735, and GSE62452. **(E)** Difference in mRNA expression of *CARD9* between normal and cancer tissues in data sets GSE15471, GSE28735, and GSE62452. **(F)** Difference in mRNA expression of *FERMT1* between the cancer cell line and normal cell line. **(G)** Difference in mRNA expression of *IL20RB* between cancer cell lines and normal cell line. **(H)** Difference in mRNA expression of *MET* between the cancer cell line and normal cell line. **(I)** Difference in mRNA expression of *MMP3* between cancer cell lines and normal cell line. **(J)** Difference in mRNA expression of *CARD9* between cancer cell lines and normal cell line (*p < 0.05, **p < 0.01, ***p < 0.001).

The expressions of *FERMT1*, *IL20RB*, *MET*, and *MMP3* were over expressed in PC samples when compared with those in normal pancreatic tissues in the cohorts GSE15471, GSE28735, and GSE62452 (*p* < 0.05) (Figures 13A–D). The expression of *CARD9* was lower in tumor tissues than it was in the normal groups for GSE28735 and GSE62452 (*p* < 0.05) cohorts. In the GSE15471 cohort, the expression of *CARD9* was higher in tumor tissues but with no statistical significance (Figure 13E).

The expression of FERMT1, IL20RB, and MET increased in most PC cell lines (CFPAC-1 and CAPAN-1) (*p* < 0.05) (Figures 13F–H). Chiefly, the expression levels of MMP3 and CARD9 were decreased in PC cell lines (MIA-PaCa-2 and CAPAN-1) (*p* < 0.05) (Figures 13I, J).

## Discussion

Recently, increasing evidence has shown a close relationship between PC and TME. The stroma and PC cells dynamically cooperate and promote all aspects of aggressiveness. From a genetic perspective, growth, metabolism, homeostasis and proliferation of cancer cells and the synthesis of cancer-related proteins are influenced by tumorigenesis, which is promoted by the mutation of genes in PC cells, such as *KRAS*, *TP53*, *CDKN2A*, and *SMAD4* (Saiki et al., 2021). At the same time, PC is very heterogeneous because it contains abundant ECM and various stromal cells, such as TAMs, MDSCs, CAFs, immunocytes, and PSCs (Reyes-Castellanos et al., 2022). Interestingly, cancer cells can work together with the TME by rewiring metabolism, autophagy, and other mechanisms to serve as vital mediators of PC progression (Perera and Bardeesy, 2015; Qin et al., 2020; Reyes-Castellanos et al., 2020; Reyes-Castellanos et al., 2022). Generally, cancer cells and the TME can promote the development, invasion, metastasis, drug resistance, and evasion of immune surveillance in PC. Previous studies have developed histological or molecular classifications to predict the prognosis of PC, but their predictive ability for therapeutic management is poor (Reid et al., 2013; Bailey et al., 2016; Gutiérrez et al., 2021). Consequently, we analyzed the genetic characteristics involved in the TME and developed a new method to predict the prognosis and sensitivity of patients to treatments in PC.

By univariate and multivariate Cox and LASSO analyses, we established a robust risk model to predict the survival of PC patients and verified it in three cohorts. Cohorts TCGA-PAAD, GSE57495, and CPTAC were used to check the practicality and reliability. Overall, the survival analysis showed that our model is helpful in identifying patients who might suffer from poor prognosis. In cohort TCGA-PAAD, the best effect of the model was exhibited at prediction for 5-year survival rate, while in cohort GSE57495, it was at 1-year. This difference may be caused by the distinction of the constitution of each cohort of patients’ clinical features. For instance, cohort GSE57495 completely focused on the early stage of PC patients. In addition, in cohort CPTAC, no patient lived more than 5 years, which resulted in the absence of AUC at 5 years. Furthermore, along with the construction of the risk model, we spotted five genes that showed an intimate relationship with the prognosis of PC patients. *MET* is a well-known gene that acts as a growth factor under physiological conditions and can promote oncogenesis via the active mode (Comoglio et al., 2008; Nakamura et al., 2011). In head and neck squamous cell carcinoma, over half of the patients overexpress *HGF* which stimulates *MET* to induce the proliferation of cell cycle genes by activating *STAT3* in the TME (Igelmann et al., 2019; Boschert et al., 2020; Raj et al., 2022). Our study found *MET* overexpressed in PC cells and tissues and high-expressions of *MET* was correlated with worse prognosis. A previous study has confirmed that *MET* facilitates stromal rewiring by upregulating tenascin-C (*TNC*) expression, which interacts with ECM components and is deeply involved in the metastasis of cancer, stimulating the proliferation and restraining the differentiation of CSCs in PC (Jones and Jones, 2000; Orend, 2005; Orend and Chiquet-Ehrismann, 2006; Modica et al., 2021). By proteolysis, *MMP3*, another high-expression gene in PC samples, destroyed various molecules, such as ECM and adhesion molecules, and enabled the tumor to be more aggressive (Sternlicht et al., 1999; Munhoz et al., 2010; Niland et al., 2021). Upregulated *MMP3* participates in the progression of genomic instability in tumors (Sun et al., 2014). By manipulating the ECM, *MMP3* is involved in oncogenesis, cancer cell proliferation, and invasion, and this explains the factor of poor survival in PC (Hadler-Olsen et al., 2013). Studies have also confirmed that the expression of MMPs is regulated by the ECM and immune system which are absent in cells *in vitro* (Conlon and Murray, 2019). This may explain the different expressions of MMP3 in PC cells and samples. Due to TGFβ signaling, the expression level of *FERMT1* mRNA increases in several PC cell lines and promotes migration and invasion (Sin et al., 2011). *CARD9* is critically involved in various inflammatory responses. By manipulating inflammatory cytokines, *CARD9* is involved in adaptive immunity (Liu et al., 2022). In cancer, the cellular location of *CARD9* is in tumor-infiltrating macrophages rather than in cancer cells (Zhong et al., 2018; Zhong et al., 2019), which explains the lower expression of CARD9 in PC cells and samples than in normal pancreatic ones. Furthermore, as determined by the biological state, macrophages, as a constituent of the TME, can boost or suppress the proliferation and metastasis of cancer (Pan et al., 2020; Xu et al., 2020; Zhou et al., 2020). This may account for the dual functions of *CARD9* that is a tumor promoter and/or tumor inhibitor (Yang et al., 2007; Yang et al., 2008; Bergmann et al., 2017; Haas et al., 2017). By working with cytokines and ECM, five genes participate in the progress of PC. In addition, this close relationship of genes and the TME in cancer makes a promising prospective prediction of survival in PC patients, and a comparison with other models certified the efficiency of our model. Next, to predict survival in a more functional and simpler way, the nomogram was established. The result of the nomogram showed that although other clinical factors were taken into account, the risk score still played the leading role.

GSEA, immune cell infiltration, and single-cell analysis confirmed that low-risk patients have a strong relationship with the immune system. The pathway enrichment analysis showed that cancer-related pathways, such as pathways in cancer and the cell cycle, were significantly enriched in the high-risk group, illustrating poor survival. However, in the low-risk group, we noticed the enrichment of immune-related pathways and this was in accordance with the result that the anti-cancer immune cells, such as T cells, CD8^+^ T cells, CTL, and B cells, were mostly infiltrated in the low-risk group. A previous study has demonstrated that better DFS and OS were guaranteed in patients by a higher expression of T cells (Muller et al., 2022). CD8^+^ T cells attack tumor cells by recognizing the antigen peptides on their surface (Fukunaga et al., 2004; van der Leun et al., 2020). The role of B cells in PC is still ambiguous. Preclinical evidence have verified that immuno-suppressive B cells could promote cancer by suppressing the activity of CD8^+^ T cells and secreting cytokines (Gunderson et al., 2016; Pylayeva-Gupta et al., 2016). However, this kind of B cells only account for 10% in B cells in PDAC in humans (Delvecchio et al., 2022). Most B cells form TLS generate an inflammatory phenotype that facilitates the activation and recruitment of antigen-presenting cells and dendritic cells (DCs) (Ene-Obong et al., 2013; Watt and Kocher, 2013; Affara et al., 2014). Therefore, the pro-tumoral role of B cells may be overwhelmed by the anti-tumorigenic role. An intimate relationship with the immune mechanism may explain better outcomes in patients with low risk. In addition, drug sensitivity analysis manifested patients gotten low risk score might benefit from the immune treatment, while the counterpart might not.

Derived from the model, we identified several potential immune targets, such as *LAG3*, *TIGIT*, and *CTLA-4* (Chihara et al., 2018; DeLong et al., 2019) that are highly expressed in the low-risk group. In PDAC, the *CD155/TIGIT* axis maintains immune evasion. Combining regimens, with inhibitors of *TIGIT* and *PD-1* plus *CD40* agonism, in preclinical models exhibited encouraging tumor suppression (Freed-Pastor et al., 2021). In the TME, T-cell exhaustion is caused by *LAG-3* that cooperates with a pile of blockade receptors (Chihara et al., 2018; Edwards et al., 2018; Karlsson et al., 2020). In mice, treatment with anti-*LAG-3* and anti-*PD-1* antibodies also showed a strong anti-tumor effect (Woo et al., 2012). Although the mentioned targets are still stuck with preclinical trials, our model suggests that immune therapy could be a promising treatment for PC patients.

Mutation of *KRAS* is one of the most common alterations in both high- and low-risk groups, being present in approximately 90% of PC patients, and is regarded as the major genetic initiating event in oncogenesis by influencing the TME and cell proliferation, apoptosis, autophagy, and metabolism (Bailey et al., 2016; Chan-Seng-Yue et al., 2020; Pereira et al., 2022). Though there was no significant difference in sensitivity of anti-*KRAS-G12C* in the two groups, we noticed patients marked with high risk are more sensitive to this drug than those at low risk. In addition, the result of GSEA in hallmark data sets showed pathway “*KRAS* signaling up” was mostly enriched in the high-risk groups. Meanwhile, considering that the mutation of KRAS *G12C* is less frequent than the mutation of *G12D* in PC, *KRAS G12D* inhibitor may be a better choice for high-risk patients.

Another gene, *ARID1A*, was only mutated in the high-risk group. The function of *ARID1A* is complicated. In colorectal cancer (CRC), the proliferation of *KRAS*-mutated cancer cell rely on *ARID1A* (Sen et al., 2019). In gynecologic cancers, *ARID1A* suppresses cancer via co-operating with p53 (Guan et al., 2011). In PC, we found that high-risk patients exhibited more *ARID1A* mutation, but the reason for this phenomenon was not clear. Anti-*ARID1A* might give us a new therapeutic target for PC. Our study identified that co-mutations of *TP53* and *KRAS* might cause worse survival. This is consistent with a former study (Shoucair et al., 2022). A preclinical study confirmed that mutated *KRAS* and *TP53* could upregulate *FOXA1* by stimulating *CREB1* and finally exert an oncogenic effect (Kim et al., 2022).

Gemcitabine plus paclitaxel and FOLFIRINOX are the first recommended chemotherapy agents for PC chemotherapy, and using our model, doctors can choose sensitive chemotherapy for PC patients (Jameson et al., 2019; Robatel and Schenk, 2022). The compound regimen of other treatments and immunotherapies, which remodels the TME by adjusting the quantities and type of T cells, exhibits promising effectiveness (Kole et al., 2022). The results verified that low-risk patients would exhibit a better response to immunotherapy. The predictive value of drug responses was weakened by the rough risk characteristics classification. As the only distinctive standard, the assessment of the quantified risk scores for evaluating drug responses cannot conclude with a precise regime. Finally, we identified several new treatment targets, but these require further substantiation in future.

To a certain extent, the use of only online databases for data verification and histological validations at a single level caused validation limitation of our model. In future, local normal and cancer pancreatic tissues and clinical data are called for to verify the robustness of our model. Meanwhile, further experiments that include genomic or proteomic analysis are demanded to investigate the mechanism of *FERMT1*, *IL20RB*, *MET*, *MMP3*, and *CARD9* in the progress of PC and verify potential treatment targets.

## Conclusion

In this study, we comprehensively analyzed the expression and prognostic value of TME-related genes in PC. We established a risk model showing high-risk patients with worse prognostic tendencies. In addition, based on this model, multiomics methods were used to explore the immune and genetic conditions to define the traits of the TME, to identify novel treatment targets (*LAG3*, *TIGIT*, and *ARID1A*) and predict diverse treatment sensitivities (high-risk patients were more resistant to oxaliplatin, irinotecan, and immunotherapy).

## Figures and Tables

**Table:** 

**PC**	Pancreatic cancer
**TME**	Tumor microenvironment
**CAFs**	Cancer-associated fibroblasts
**PSCs**	Pancreatic stellate cells
**ECM**	Extracellular matrix
**TAMs**	Tumor-associated macrophages
**MDSCs**	Myeloid-derived suppressor cells
**Tregs**	Regulatory T cells
**EMT**	Epithelial–mesenchymal transformation
**CSCs**	Cancer stem cells
**TILs**	Tumor-infiltrating lymphocytes
**DFS**	Disease-free survival
**OS**	Overall survival
**MSI-H**	High microsatellite instability
**dMMR**	Mismatch repair deficiency
**TMB**	Tumor mutational burden
**PAAD**	Pancreatic adenocarcinoma
**TCGA**	The Cancer Genome Atlas
**GEO**	Gene Expression Omnibus
**FPKM**	Fragments per kilobase of exon model per million mapped fragments
**CPTAC**	Clinical Proteomic Tumor Analysis Consortium
**RSEM**	RNA sequencing by expectation-maximization
**RMA**	Robust multichip average
**MSigDB**	Molecular Signatures Database
**NMF**	Non-negative matrix factorization
**LASSO**	Least absolute shrinkage and selection operator
**RS**	Risk score
**AUC**	Area under the curve
**ROC**	Receiver operating characteristic
**K-M curves**	Kaplan–Meier curves
**C-index**	Harrell’s concordance index
**DCA**	Decision curve analysis
**PPI**	Protein–protein interaction
**GSEA**	Gene set enrichment analysis
**COSMIC**	Catalogue of Somatic Mutations in Cancer
**CNVs**	Copy number variations
**FOLFIRINOX**	5-Fluorouracil, oxaliplatin, irinotecan, and leucovorin)
**IC50**	Half-maximal inhibitory concentration
**GEP**	T-cell inflamed gene expression profile
**CYT**	Cytotoxic activity
**TIDE**	Tumor Immune Dysfunction and Exclusion
**IMvigor210**	Immunotherapeutic cohort
**RECIST**	Response evaluation criteria in solid tumors
**CR**	Complete response
**PR**	Partial response
**SD**	Stable disease
**PD**	Progressive disease
**C1/2**	Cluster 1/2
**CTLs**	Cytotoxic lymphocytes
**NK**	Natural killer cells
**mDCs**	Myeloid dendritic cells
**SMGs**	Significantly mutated genes
**SNVs**	Single-nucleotide variations
**SNP**	Single-nucleotide polymorphism
**KEGG**	Kyoto Encyclopedia of Genes and Genomes
**TNC**	Tenascin-C
**NES**	Normalized enrichment score

## Data Availability

The data are available from the TCGA (https://portal.gdc.cancer.gov/), GEO (https://www.ncbi.nlm.nih.gov/geo/), and cBioportal (https://www.cbioportal.org/). The accession numbers can be found in the article/supplementary material.
