# Supplementary figures and images for: Prognostic utility of TME-associated genes in pancreatic cancer
Source: Front Genet. 2023 Sep 1;14:1218774. doi: 10.3389/fgene.2023.1218774 (PMC10505756; doi:10.3389/fgene.2023.1218774)

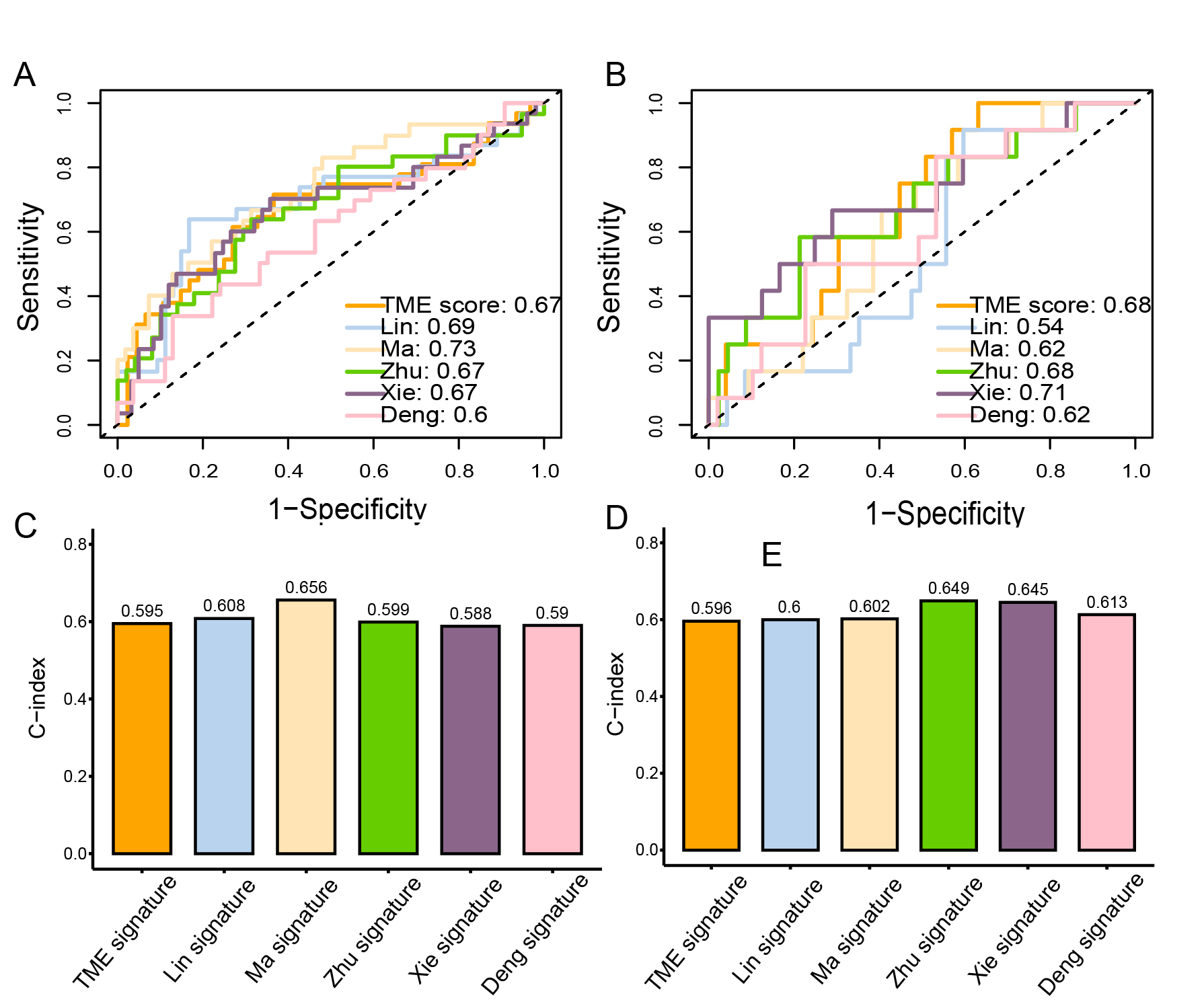

Supplement: Supplementary file 1 [file DataSheet1.zip › Data Sheet 1/Supplementary Figure S1.jpg]

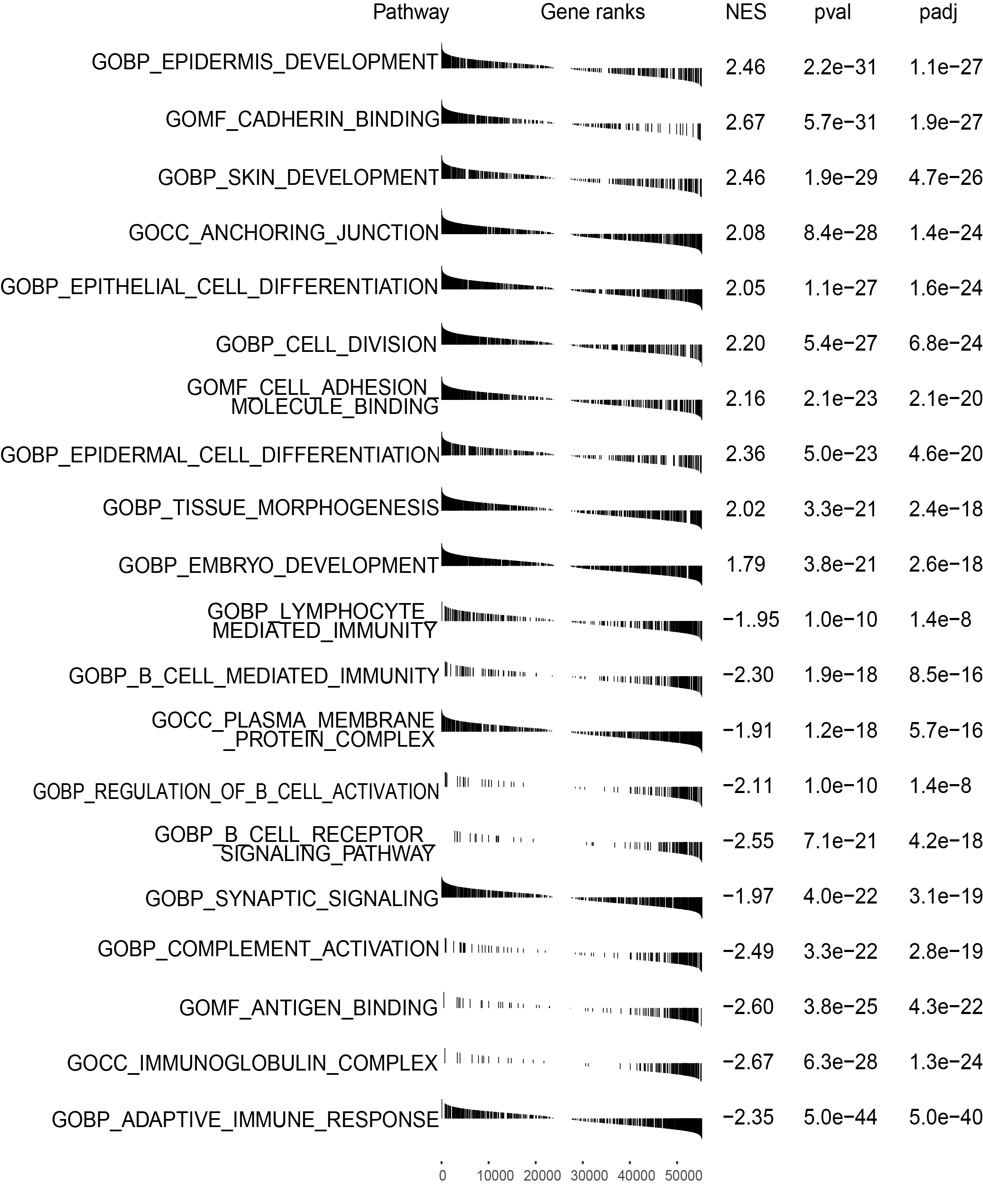

Supplement: Supplementary file 1 [file DataSheet1.zip › Data Sheet 1/Supplementary Figure S2.jpg]
